# Acknowledgment to Reviewers of *Polymers* in 2021

**DOI:** 10.3390/polym14030473

**Published:** 2022-01-25

**Authors:** Polymers Editorial Office

**Affiliations:** MDPI AG, St. Alban-Anlage 66, 4052 Basel, Switzerland

Rigorous peer-reviews are the basis of high-quality academic publishing. Thanks to the great efforts of our reviewers, *Polymers* was able to maintain its standards for the high quality of its published papers. Thanks to the contribution of our reviewers, in 2021, the median time to first decision was 11 days and the median time to publication was 33 days. The editors would like to extend their gratitude and recognition to the following reviewers for their precious time and dedication, regardless of whether the papers they reviewed were finally published:
Abbas KhoshnoodLidija Slemenik PerseAbdelghani BenyoucefLilia SabantinaAbdelhamid ElaissariLiliya S. LogoydaAbdel-Hamid Ismail MouradLilly LiAbdelkader KahouliLin LiAbdelsamed ElshamyLin SangAbdul AhadLin XiaoAbdul KhanLingxin ChenAbdul Samad MohammedLiqun NingAbdul-Sattar NizamiLiqun ZhangAbdussalam QaroushLi-Ting LeeAbhilash VenkateshaiahLiutauras MarcinauskasAbhishek GhoshLiyuan ShengAbolfazl MohebbiLjerka Kratofil KrehulaAbu Zafar Al MunsurLong Chiau MingAda-Ioana BuneaLongteng TangAdam EkielskiLorenza DraghiAdam GnatowskiLorenzo MinoÁdám JuhászLourdes FrancoAdelaide MirandaLu AnAdeolu Adesoji AdediranLu HanAdraino GalvaoLu ShaoAdriaan S. LuytLubos KristakAdrian Alexandru SerbanoiuLuca Michele MartulliAdrian AntosikLucia BaldinoAdrian KellerLucia CatucciAdrián MatencioLucia MontenegroAdrian NeaguLucia MorbidelliAdrián RojasLuciano BoeselAdrian Saura-SanmartinLucjan KozielskiAdriana BalanLuigi BennardoAdriana Burlea-SchiopoiuLuigi PigaAdriana KovalcikLuigi TodaroAdy SuwardiLuis A. MiccioAfonso Rangel Garcez De AzevedoLuis Alfonso Trujillo-CayadoAgenor Valadares SantosLuís BernardoAgnė KairytėLuís Cláudio Nascimento da SilvaAgnieszka A. PilarskaLuis Felipe De Paula SantosAgnieszka Ewa Wia̧cekLuis Garzón-TovarAgnieszka Gadomska-GajadhurLuis Jesús Villarreal-GómezAgnieszka J. NowakLuís PinhoAgnieszka Kijo-KleczkowskaLuis Rodríguez-LorenzoAgnieszka KowalczukLuisa CarvalhoAgnieszka KowalczykLuiz Carlos BertolinoAgnieszka KyziołLuiza MercanteAgnieszka MarcinkowskaŁukasz SadowskiAgnieszka TercjakŁukasz SzeleszczukAgustín Lara-SánchezLuminita DumaAgustin RasconLuminita MarinAhmad JabbarzadehLung-Chien ChenAhmad MoghimikheirabadiLutfor RahmanAhmed A. GheniLuz Fernanda Santos daAhmed ElkaseerLyazid BouhalaAimeric OuvrardM. R. MozafariAiqin WangMaarten MeesAira MatsugakiMaciej GryszelAirat SakhabutdinovMaciej GuzikAkihiko FujiwaraMaciej Łukasz DębowskiAkihiko IshiiMaciej SkotakAkihiro NakataniMaduka Lankani WeththimuniAkihito HashidzumeMagdalena BrodaAkilarasan MuthumariappanMagdalena Cristina StanciuAlaa AdawyMagdalena Klimek-OchabAlaeddin Burak IrezMagdalena KwiatkowskaAlain Tundidor-CambaMagdalena LaskowskaAlan TaubMagdalena MaciejewskaAlbert LeeMagdalena SobiesiakAlbert PoaterMahdi BodaghiAlberto ConcellónMahdi RezapourAlberto García-PeñasMahdieh ShahmardaniAlberto GregoriMahendran SekarAlberto RomeroMahzan JoharAldobenedetto ZottiMajid AliAlejandro BugarinMakoto HasegawaAlejandro J. AlvarezMaksym PogorielovAlejandro Presa SotoMakusu TsutsuiAlejandro Ramírez-JiménezMałgorzata GniewoszAlejandro Vega–RiosMałgorzata MatusiakAleksandar MehandzhiyskiMałgorzata MusiałAleksandar Ž. KostićMałgorzata Szultka-MłyńskaAleksander HejnaManfred DunkyAleksandra TutuevaManickam MinakshiAleksandra V. BukhovetsManjunath ShettarAleksey D. DrozdovManoli M. ZubiturAlena SiskovaManoli ZubiturAlenka VeselManuel BlázquezAleš MizeraManuel Ferrandez-VillenaAlessandra LuccheseManuel JordánAlessandro F. MartinsManuel Salado ManzorroAlessandro PatelliManuel Viuda-MartosAlessandro ScarsoMar Fernández-GutiérrezAlessandro TorchioMara CamaitiAlex Elías-ZúñigaMarcel F. KunrathAlexander FedorchenkoMarcelo AntunesAlexander MalkinMarcia MeierAlexander MünchMarcin BorowiczAlexander NguyenMarcin JesionekAlexander NovikovMarcin LemanowiczAlexander PogrebnjakMarcin StruszczykAlexander SidorenkoMarcin WekwejtAlexander ToikkaMarcin WłochAlexandra BarganMarcin WysokowskiAlexandra PulyalinaMarco AlfanoAlexandra ShakunMarco Antonio Garza NavarroAlexandre SerresMarco CamperaAlexandros K. NikolaidisMarco GovoniAlexandru Mihai GrumezescuMarco MartinoAlexey BeskopylnyMarco MorraAlexey BobrovskyMarco PaolinoAlexey BubnovMarco SacchiAlexey ChubarovMarco SalernoAlexey IordanskiiMarco SerraAlexey SalimonMarcos F. S. TeixeiraAlexey V. LyulinMarcos Rodríguez MillánAlfredo GüemesMarcus BaumannAlfredo Vázquez-OvandoMareike WolterAli AltwayMarek IsbrandtAli Farokhi NejadMarek MackoAli Ibrahim Al-MosawiMarek NowickiAli KandemirMarek PokornyAli Reza ZanjanijamMarek PolanskiAli Salehzadeh-YazdiMarek SzkodoAli SalemMarek SzostakAlice MijaMari Cruz García-GutiérrezAlina AdamsMari De MeijerAlina ButuMaria AtanassovaAlireza SassaniMaria Cristina RighettiAlireza TabatabaeiMaria Daniela StelescuAlovisi MarioMaria De La Luz ZambranoÁlvarez Pablo PujadasMaria DergachevaAlwar RamaniMaria Dolores La Rubia GarciaAmaia Soto BeobideMaría Elena Sánchez-VergaraAmarajothi DhakshinamoorthyMaria Erminia GenoveseAmid HoomanMaria Francesca Di FilippoAmin MojiriMaria HarjaAmin ShavandiMaria Helena CasimiroAmir Masoud PourrahimiMaria Helena FernandesAmirhossein MadadiMaría Isabel Rial-HermidaAmirreza KhodadadianMaria J. SuarezAmod A. OgaleMaria LeżańskaAna AlcudiaMaria Luisa TorreAna Beltrán SanahujaMaria MolinaAna Catarina SousaMaria NetoAna CazacuMaria NikolovaAna De SousaMaria NowackaAna FonsecaMaria P. SokolovaAna HorovistizMaria PantanoAna López De DicastilloMaria Paola LudaAna M. Carmona-RibeiroMaria Petrovna SokolovaAna Maria de Matos CharasMaria RaimoAna María Díez-PascualMaria RapaAna Paula Betencourt Martins AmaroMaria SpaneddaAna Paula PiedadeMaria ZieleckaAna PilipovićMariacecilia PasiniAna Rita Costa-PintoMarialuigia RaimondoAnandram VenkatasubramanianMariamelia StanzioneAnastasios GotziasMarian Gabriel BanciuAnastasiya SolovievaMarián LehockýAnca DinischiotuMariana CristeaAnca LascuMariana S. CastroAnca Niculina CadinoiuMariano RomeroAncuţa Elena TiucMariano VenanziAnderson LoboMariateresa LettieriAndré Luiz MissioMarija StojmenovićAndré Moreni LopesMarijana JurićAndré Rolim BabyMarijo BuzukAndrea AbateMarilyne RoumanieAndrea ArsiccioMarin MicutzAndrea CochisMarin SenilaAndrea DoderoMarina ArrietaAndrea DorigatoMarina LambertiAndrea EhrmannMarina MatosAndrea PaolellaMarina P. ArrietaAndrea ProtoMarina PaolucciAndrea RagusaMarina RuthsAndrea SpagnoliMarina VukojeAndrea SpitaleriMarinos PitsikalisAndrea ZilleMario CalveteAndreas BückMario E. FloresAndreas KandelbauerMárió GajdácsAndreas MautnerMario VersaciAndreas RosenkranzMariola KozłowskaAndreea-Teodora IacobMariola RajcaAndrei IvanetsMarissa PaglicawanAndrei PotapovMarius-Andrei OlariuAndrei SarbuMariusz FabijańskiAndrei Vasile NastutaMariusz KosobudzkiAndres Sanz GarciaMariusz MaminskiAndrew B. CrollMariusz TryznowskiAndrew NaylorMariya SpasovaAndrew PikeMark JacksonAndrey KrauklisMark LowryAndrzej AmbroziakMarko KrstićAndrzej BąkMarko PetricAndrzej DzierwaMarkos PetousisAndrzej KasperskiMarkus EuringAndrzej KatuninMarkus GahleitnerAndrzej NastajMarkus StommelAndrzej PuszkaMarta ArczewskaAndrzej SwinarewMarta C. P. C. CorvoAndrzej TomporowskiMarta Fiedot-TobołaAneta KaniaMarta GrochowiczÁngel Fernández CuelloMarta HarničárováAngel R. Hernandez-MartinezMarta MusiołAngela De BonisMarta SzekalskaAngela LongoMarta Tsirigotis-ManieckaAngela MalaraMarta WorzakowskaAngela ScalaMartha Verónica Escárcega BobadillaAnicuta StoicaMartin BaumgartenAniket M. RishiMartin DankoAnil K. BastolaMartin DunnAnita TarbukMartin GerickeAnja BarabaMartin KollerAnju GuptaMartin KozakAnka Trajkovska PetkoskaMartin SahulAnkica ŠarićMartins KleinhofsAnkit K. RochaniMarwa ElkadyAnkur BajpaiMarwa TallawiAnna DrabczykMaryam Mobed-MiremadiAnna KusiorMarzena PawlikAnna MasekMarzio GrassoAnna MertasMasahiro OkadaAnna Sroka-BartnickaMasao IrieAnna SzwajcaMasao TamadaAnna SzymczykMasashi IkegamiAnna TrusekMasataka KuboAnna WcisłoMasayuki YamaguchiAnna Zimoch-KorzyckaMashaalah ZarejousheghaniAnna-Liisa PeikolainenMasoumeh OzmaianAnnalisa ChiapponeMassimo DuranteAnnalisa PaoloneMassimo LazzariAnnalisa VolpeMassimo OttonelliAnnamaria ViscoMateusz KoziołAnne AndersonMatheus PolettoAnne Schwarz-PfeillerMatteo BaggioliAnroop NairMatthew DerryAnthony DichiaraMatthew PanAnthony KotulaMattia BartoliAntolino Gallego MolinaMattia RapaAnton BonartsevMatúš MolčanAntón Civit-BalcellsMaurice CollinsAnton ManakhovMaurizio ArenaAnton MostovoyMauro CarraroAnton V. DolzhenkoMauro FilippiniAntonella Caterina BocciaMauro ZarrelliAntonella PattiMavinkere Rangappa SanjayAntonella PiozziMaxim GorkunovAntonino S. FiorilloMazeyar Parvinzadeh GashtiAntonino ValenzaMaziar HeidariAntonio ApicellaMeaurio EmilianoAntonio CaggianoMehmet KodalAntonio CapezzaMehmet SenelAntonio ConcilioMehmet YilmazAntonio Di BartolomeoMehrez E. El-NagarAntônio Gilson Barbosa de LimaMelanie EckerAntonio GrandeMelkie Getnet TadesseAntonio GrecoMenake PiyasenaAntonio LamuraMeng ZhangAntonio PizziMengfang LiAntonio SancezMengmeng LiuAntonio Torres MarquesMengyuan ChenAntonio VittoriaMercedes JiménezAntonio ZuorroMh Busra FauziAntonios N. PapadopoulosMichael ArkasAntonis GitsasMichael BertocchiAntoniya TonchevaMichael ChaeAntony AnanthMichael CiesielskiAnuj KumarMichael FeuchterAnurag PisupatiMichael GradyAnzar KhanMichael HauptAparna BanerjeeMichael I. OjovanAránzazu MendíaMichael IoelovichArash KarimipourMichael MeyerAratz GenuaMichael R. KoblischkaAravin Prince PeriyasamyMichael Rudolf Koblischka Araz Rajabi-AbhariMichael SchmittAref Samadi-DookiMichael Yin WongAriane RozzaMichail KlontzasArif U. AlamMichał BöhmArindam ChakrabartyMichal BoreckiAris GiannakasMichal DudekAristotelis SgourosMichał DutkiewiczArkadij SobolevMichal PetruArmando Roman-FloresMichal PuskarArnaldo Leal-JuniorMichal SedlačíkArnaldo Rodrigues Santos JúniorMichele CaraglioArnaud BertschMichele FerrariArtem EreminMichelle SalviaArtem KovalenkoMichinari KohriArtur J. M. ValenteMiguel A. OrtegaArtur TurekMiguel AlcarazArun Kumar TeotiaMiguel Angel OrtegaArvind GuptaMiguel Torres-RodríguezAsad HanifMihaela BaibaracAsandulesa MihaiMihaela GirtanAsit GainMihaela HomocianuAtefeh KarimzadeharaniMihai BoniAthanasios KungolosMihai BrebuAtsushi MorikawaMihai SăndulescuAtsushi SakamotoMihalj PoŝaAtsushi SudoMihkel KoelAtul SharmaMika SalmiAugusto Cannone FalchettoMike BambachAurel DiaconMikhail M. MaslovAurelia MegheaMikhail M. Vorob'evAurélie TaguetMikhail N. KrakhalevAvinash BajiMikhail ShamoninAxel T. NeffeMikihito TakenakaAyachi SahbiMiklós NagyAyae Sugawara-NarutakiMikyung ShinAzimi Yancheshme AmirMilad PourbaghiBahareh AzimiMilad SaeedifarBaki HazerMilada GajtanskaBangjing LiMilan ŽmindákBarbara CerroniMilena KušnerováBarbara HajdukMilica SpasojevicBarbara HinterstoisserMiljana MirkovićBarrio Jesús DelMiloš PánekBartłomiej KostMin ParkBasim AbujdayilMin Wook LeeBeata KurcMinas M. StylianakisBeata Morak-MłodawskaMingxing LiBeata PodkoscielnaMingze SunBeata StrzemieckaMinjee KangBeata Świeczko-ŻurekMinkook KimBela KocsisMiran MozetičBelen BeginesMircea BeuranBen Bin XuMircea ChiparaBenoît HeinrichMirela-Fernanda ZaltariovBeom Jin KimMirjana KostićBernadette Emoke TelekyMirjana Pejić BachBernardeta DębskaMiroslav MaňasBernhard SchmidtMiroslav MensikBettina WolfMiroslava Dušková-SmrčkováBianca ManigliaMirosław KwiatkowskiBijender KumarMirta I. ArangurenBiljana GlišićMiruna StanBin LiMitja KolarBin YuMitsuru AbeBing-Hung ChenMohamed A. HassanBipin GaihreMohamed B. ZakariaBishweshwar PantMohamed BassyouniBlaž LikozarMohamed El-NewehyBogdan IstrateMohamed H. MussaBogdan ŞerbanMohamed Lotfy AshourBogdan-George RusuMohammad Afsar UddinBohuslava TremlováMohammad AhmadiBojan ČalijaMohammad Ali HesarinejadBolesław SzadkowskiMohammad AlotaibiBon-Cheol KuMohammad Javed AnsariBorislav AngelovMohammad K. HassanBosko RasuoMohammad Mizanur RahmanBożena Katarzyna ZgardzińskaMohammad Reza SafaeiBozhidar StefanovMohammad Sayem MozumderBrahmananda PramanikMohammad Tavakkoli YarakiBrian K. ViaMohammad Zahirul IslamBriliant Adhi PrabowoMohammadreza NaeimiradBruno Henrique VilsinskiMohammadreza ShokouhimehrBryan BeckinghamMohammed Nadeem BijleBryan WongMohammed Rafi ShaikBudimir MijovićMohammed SabbahBurak GerisliogluMohammednoor AltarawnehBystrík DolníkMohammednoor Khalil AltarawnehCamelia CerbuMohan Kumar Muthu KaruppanCamelia VizireanuMohd Bijarimi Mat PiahCamilo Andres FrancoMohd Nurazzi NorizanCan JiangMohd Sapuan SalitCan LiuMohd Shaiful SajabCarl TrindleMohd Usman Mohd JunaidiCarlo CastellanoMohd Zamidi AhmadCarlo PastoreMohd Zuhri Mohamed YusoffCarlo PaternosterMohsen Beladi MousaviCarlo SantulliMokarram HossainCarlos Alberto Antunes ViegasMokgaotsa MochaneCarlos Alberto Ferreira MarquesMona SemsarilarCarlos David Grande TovarMonica BertoldoCarlos Eduardo Fonseca-AlvesMonica FuensantaCarlos F. O. GraeffMonica OliveiraCarlos Guerrero SanchezMonika Kadela-TomanekCarlos LunaMonika SommerhalterCarlos MarquesMonoj GhoshCarlos MenesesMoon-Sung KangCarlos Miguel MartoMoreno MarcelliniCarlos Rivera-GómezMorshed KhandakerCarlos SalasMorteza KafshgariCarlos ScuracchioMosab KaseemCarly S. FilgueiraMotahira HashmiCarmelo CorsaroMounir ChaouchCarmen Alice TeacǎMourouzis PetrosCarmen GaidauMudassir HasanCarmine CapacchioneMuhammad AamirCarol BarryMuhammad Ahsan SaeedCarson J. BrunsMuhammad AkbarCatalin PruncuMuhammad Amber FareedCatalin ZahariaMuhammad Asyraf Muhammad RizalCatalina Natalia Cheaburu YilmazMuhammad BilalCatarina S. H. JesusMuhammad Imran MalikCatherine KellyMuhammad IqbalCatia AlgieriMuhammad Kashif ShahidCécile Formosa-DagueMuhammad Mubashir Bhatti Cédric DelattreMuhammad MujtabaCélio Bruno Pinto FernandesMuhammad SaleemCerezo Maria Natividad GomezMuhammad Shahzad KamalCésar S. HuertasMuhammad Sohail ZafarCesare CamettiMuhammad Tayyab NomanChai Hoon KooMuhammad Usman GhoriChai-Lin KaoMuhammad Wajid UllahChan I. ChungMuhammad ZafarChanchal Kumar KunduMuhammad Zaheer SaleemChandan GiriMuhammed Naseem AkhtarChander PrakashMuhibUr RahmanChandrashekhar VoshavarMunirah Binti Sha'BanChang Kook HongMurat DemiralChanggu LeeMuruganandan ShanmugamChanghai ChenMurugesan ChandrasekaranChan-Woo ParkMusuc Adina MagdalenaChao WangMuthaiah ShellaiahChao XuMuzamil KhatriChao-Tsai HuangNadeem BaigChao-Wei TangNader AsnafiCharles BrennanNader ShehataChen FangNadia MartuccielloCheng HeNanang MasruchinCheng ZhangNaoya AdachiChenggao LiNaozumi TeramotoCheng-Ho ChenNaresh KasojuChengmin JiangNarmidi MamidiCheng-Ming TangNaser KabashiChenxi BaiNasim NosoudiCheol-Hong HwangNatalia Ivanovna KozhukhovaCheuk Lun LiuNatalia Moreno-CastellanosChiachung ChenNataliya A. SakharovaChia-Her LinNatalya A. KulikovaChia-Hung Dylan TsaiNataša Z. TomićChiara MartinelliNathalie MarlinChien-Hong LinNathalie Zink LorreChien-Lin HuangNavendu JanaChih Chiang HongNavideh AbbasnezhadChih-Feng HuangNeal HickeyChih-Hao LeeNectarios VidakisChin Lung ChiangNeith PachecoChing Hao LeeNejib KasmiChirag GodiyaNektarios KalyvasChiwai KanNeli KosevaChristian BraunerNemanja GavrilovChristian KuklaNemany Abdelhamid Nemany HanafyChristian-Alexander BungeNemany HanafyChristina KaravasiliNgoc A. NguyenChristopher G. HuntNgoc Hung VuChristopher GibsonNicanor CimpoesuChristopher N. LaFrattaNicola ZampieriChristopher P. GreenNicolas LebonChristos K. KontosNicoletta DitarantoChristova DarinkaNicu BizonChrysanthos MaraveasNidhin DivakaranChuanyin XiongNieves López-SalasChuanyu SunNikolai TsvetkovChulhwan ParkNikolaj MoleChun-Chi ChenNikolaos NazirisChung-Sun LimNikolaos PolitakosChun-Kai WangNikolay GorshkovChun-Yung HuangNikos TzortzakisCindy ZhaoNikša DulčićClara Casado-CoterilloNima MovahediClara GomesNimer MurshidClaudia Maria SimonescuNina GunkelmannCodruta SarosiNirav JoshiColin BonduelleNitesh MittalColleen McMahanNoel Jacob KaleekkalColuccini CarmineNoel Peter TanConstantin ChaliorisNorbert Krisztián KovácsConstantin MircioiuNorbert NemesCorina Marilena CristacheNorio NakataCorneliu TanaseNorizah RahmanCristian M. O. LéporiNorma Aidé Cortez-LemusCristian SimionNorma-Aurea Rangel-VázquezCristian VendittozziNorman S. AllenCristiana RadulescuNubia Zuverza-MenaCristiano E. Rodrigues ReisNuggehalli M. RavindraCristiano ReisNuha MashaanCristina CazanNuria AlegretCristina MinnelliNuruzzaman NoorCristina SatrianoOctavian Mădălin BunoiuCristina StancuOdda Ruiz De BallesterosCristóbal García ParienteOgnen Pop-GeorgievskiCsaba BalazsiOh Seok KwonCüneyt H. ÜnlüOjha Gunendra PrasadDa ChenOkikiola AgbabiakaDai-Soo LeeOlaf HesebeckDaisuke KondohOleg V. GradovDaiva BaltriukieneOleg Vladislavovich LevinDamien BrunelOleksandr GryshkovDan DobrotăOleksandr KovalchukDan Mihai ConstantinescuOleksandr MolnarDan RosuOlga Evgenevna GlukhovaDani DordevicOlga KlinkovaDaniel BreiteOlga TrhlíkováDaniela BeckerOliver BrüggemannDaniela CalinaOlufunmilayo JosephDaniela IannazzoOlya StoilovaDaniela RusuOndrej JurcekDaniele BattegazzoreOnur YilmazDaniele CangialosiOriele PalumboDaniele MantioneOsamu KamiyaDaniele MassellaOscar ZambranoDaniele SilvestriOskar J. BeraDaniil NikitinOsvaldo Agustín LambriDanijela MarovićOvidiu NemesDanil PimenovOvidiu OpreaDanuta MatykiewiczOzgul GokDapeng WangOzlem Ipek Kalaoglu-AltanDarinka FakinPablo Rafael Pardo IbáñezDariusz BartkowskiPagot GioeleDariusz JarzabekPall EmokeDariusz KokoszyńskiPanagiotis StavropoulosDaryl BowmanPanayotis BenetatosDavid CabaleiroPanita MaturavongsaditDavid MillsPaola GandiniDavid MoorePaolo CapparèDavid YeboahPaolo FerrutiDavide MasatoPaolo ForaboschiDavide NocitaPaolo MarconciniDavide PapurelloPaolo S. CalabròDavide PicchiPaolo S. ValvoDawid JanasPaolo TrucilloDawid ZychPapović SnežanaDayun YanParthiban MarimuthuDebananda MohapatraPaschalis CharalampousDébora Lopes Salles ScheffelPatchiya PhanthongDechao GengPatrícia BatistaDeclan DevinePatricia Kara De MaeijerDeepti SinghPatricia Losada-PerezDelia Mihaela RaţăPatricia SeverinoDemetrio Jackson Dos SantosPatricio Orellana-PalmaDeng-Guang YuPatrick IlgDengpan DongPatrick TheatoDenis B. ZolotukhinPatrizia TrovalusciDenis Mihaela PanaitescuPatrycja MakośDenis RodriguePaul JoyceDenise BellisarioPaul SottaDerval Dos Santos RosaPaul SundaramDhamodharan VenugopalPaula BoschDharamdeep JainPaula FerreiraDi WangPaula Malo De MolinaDi YouPaula ZapataDiana AntalPaulius GriškevičiusDiana CiolacuPaulo CaldasDiannan LuPaulo J. PalmaDiego AntonioliPaulo Ricardo De SouzaDiego Romano PerinelliPavel ChapalaDiego Said SchicchiPavel MokrejšDiego VergaraPavel PadnyaDijana GrgasPavel ReitermanDiletta AmiPavel UrbánekDimitrio KontziampasisPavel V. KomarovDimitrios A. DragatogiannisPavel Y. ApelDimitrios GkiliopoulosPavle M. SpasojevicDimitrios NikolopoulosPavle SpasojevicDimitrios-Sotirios KourkoumpasPavlo BekhtaDimitris S. AchiliasPavlo MaruschakDingying ZhouPawan Kumar MishraDionysis E. MouzakisPawan MishraDipankar RoyPaweł ChmielarzDivya SharmaPaweł GłuchowskiDjalal TrachePaweł PiszkoDjordje MedarevicPaweł PtaszekDmitry A. ZimnyakovPaz Antonio Tabernero deDmitry IsakovPedro Jesús Herrera-FrancoDmitry VolodkinPedro MartinhoDo Yeung YoonPedro PradanosDobromira ShopovaPedro Raposeiro da SilvaDoina HumelnicuPei-Chun WongDolores Remedios SerranoPeng ChenDomenico AciernoPeng WuDomenico SagnelliPengtao LuDomenico TruzzolilloPengyi ZhaoDominic WalesPeng-Yuan WangDonata IandoloPer HanssonDong Jin YooPere CastellDong MengPere MutjèDongdong GePeter R. LaityDongfang ZhouPetr FilipDonghyun LeePetr FormánekDongshen TongPetr KoudelkaDong-Wook HanPetr ŠálekDoris PospiechPetr ShibaevDorna EsrafilzadehPetr SvobodaDorota DziurkaPetr SyselDorota NeugebauerPetra BujňákováDrissi-Habti MonssefPetra PeerDrozlowska EmiliaPetre IonitaDubravko RogalePetricǎ VizureanuDumitru NedelcuPetronela NechitaDumitru OlaruPhilippe LambinDuong NguyenPhilippe LecomteDurga ParajuliPietro RussoDzmitry ShcharbinPing LiEamor WooPing’an SongEdgar Franco-UrquizaPiotr BorysiukEdina RusenPiotr KurcokEdit CsapoPiotr LesiakEdson Silva FilhoPiotr LulińskiEduardo A. Lopez-MaldonadoPiotr PrzybylekEduardo GuzmánPiotr RybarczykEduardo Moreira Da SilvaPiotr RytlewskiEduardo RoblesPiyush Sindhu SharmaEdward A. EvansPolina P. KuzhirEdward BormashenkoPooria PasbakhshEfrain Mejia-BeltranPooyan MakvandiEhsan Nazarzadeh ZarePoushali DasEhtasham MustafaPo-Wen ChenElena A. SilantyevaPrabakaran MayakrishnanElena FerrettiPrabhakarn ArunachalamElena Gómez-SánchezPrabhu MathiyalaganElena PopaPradeep KumarElena SaguerPradeep R. VaradwajEleni KotsiomitiPradip DasElham Hosseini NejadPrasun KumarElias ChristoforidesPraveen ThoniyotElisa CarignaniPreeti TyagiElisa PassagliaPreety AhujaElizabeth Carvajal-MillanPřemysl LubalEllen WasanPriyanka RayElsa LasseuguettePriyanka SharmaElżbieta MegielProkopios GeorgopanosElżbieta SzostakProspero Di PierroEmanuel M. FernandesPrzemyslaw BartczakEmanuela SgrecciaPrzemysław DorożyńskiEmanuela SperanziniPrzemysław GolewskiEmi HaladjovaPrzemysław LedwońEmil PetrescuPrzemysław PączkowskiEmilia IrzmanskaPrzemyslaw SowinskiEmilia P. CollarPui-Lam NgEmiliano LaudadioPurcar VioletaEmiliano MeaurioQi RaoEmmanuel Atta-ObengQiang GuoEmmanuel LamourouxQianhui LiuEmmanuel Urandu MapesaQianqian ZhangEnrico StortiQichang MeiEnrique CasarejosQing-Fu SunEnrique Cuan-UrquizoQinqin ZhangEnsanya A. Abou NeelQiyao HuangEnshirah Da'naQuim TarrésEnzo MartinelliRachana SahneyErick S. VasquezRachele CastaldoErik CartonRachid TouzaniErika MúdraRadoslaw DrozdErjin ZhengRadostina GeorgievaErlantz LizundiaRadu-Eugen BreazEsam YahyaRaed Abu-ReziqEsfandiar PakdelRafael ComesañaEsmaeil Doustkhah HeraghRafael CuestaEstefanía Álvarez-CastilloRafael E. HincapieEster ChiessiRafael LundEsther RebollarRafał AnyszkaEugenia Fagadar-CosmaRafał BielasEuiseok LeeRafał JanuszewskiEun Young JungRafał KozdrachEunice CarrilhoRafał MalinowskiEva PiorkowskaRajan ChoudharyEvando AraújoRajan KrishnaprasadEvangelia VouvoudiRajesh MishraEvgenia Korzhikova-VlakhRaji AtchudanEwa GlowinskaRakkiyappan ChandranEwa KorzeniewskaRalph Rolly GonzalesEwa Olewnik-KruszkowskaRaluca IanchisFabia GrisiRaluca Nicoleta Darie-NitaFabia KarineRamachandran Chidambaram SeshadriFabian ItelRamanaskanda BraveenthFabien SalaünRami HawilehFabio Di NardoRamon Canela-GarayoaFábio Filipe Fereira GarrudoRamón G. RubioFabio GanazzoliRamón PamiesFabio GiudiceRamona GalatusFabio ZobiRan SuckeverieneFabricio MachadoRan TaoFabricio Maestá BezerraRania HathoutFaheem AkhtarRanjit DeFahmi ZaïriRasim AlosmanovFaiyaz ShakeelRasmina HalisFakhroddin NazariRasoul Esmaeely NeisianyFalko PippigRavindranadh KoutavarapuFan YangRawaz KurdaFang-Chyou ChiuRayya A. Al-BalushiFarayde Matta FakhouriRazvan UdroiuFarooq SherRebar AbdulwahidFarzaneh SabbaghRegina Fuchs-GodecFatemeh MokhtariRegine FrankFatima El KhalloufiRegis BarilleFatma YalcinkayaRenata JastrzabFederico AnticoRenata WłodarczykFederico BellaRené HägerlingFederico CerroneReza KolahchiFederico Vázquez HurtadoReza MalekimoghadamFedor SenatovRicardo FaccioFei BinRicardo KamikawachiFei GuoRicardo M. F. FernandesFei WangRicardo MallaviaFeipeng YangRiccardo CorpinoFelix H. SchacherRidhwan JumaidinFeng JunRima D. AlharthyFerenc RonkayRita Bacelar FigueiraFernando GalembeckRita CastroFilippo PieriniRobert CzarnotaFiore P. NicolettaRobert O. DunnFioretta AsaroRobert OpilaFirdos Alam KhanRobert PrzekopFlavio RizzolioRobert UlewiczFlora N. BalievaRobert WojcieszakFlorentina-Daniela MunteanuRoberta BertaniFlorin OanceaRoberta PeilaFrancesc EstranyRoberto AguadoFrancesc Xavier EspinachRoberto BaroneFrancesca LionettoRoberto BernasconiFrancesca LuziRoberto Castro-MuñozFrancesca ModugnoRoberto FiorenzaFrancesca RussoRoberto GramignoliFrancesco BertocciRoberto LópezFrancesco CiardelliRoberto SpinaFrancesco ClementiRobin HutchinsonFrancesco ColangeloRobin ZuluagaFrancesco Della MonicaRocco Di GirolamoFrancesco FusoRocio Canovas MartinezFrancesco LoprestiRodrigo NavarroFrancesco Paolo La MantiaRodrigo PessoaFrancesco TornabeneRodrigo Sávio PessoaFrancisca García-Moreno NisaRoger M. RowellFrancisco Fraga-LópezRogério Leone BuchaimFrancisco Javier NavarroRok FinkFrancisco SilvaRoland R. NetzFrancisco SolanoRoman FediukFranck CamerelRoman JaskulskiFrancois BauerRoman PopielarzFrank ClemensRoman SzewczykFrantišek KrčmaRoman Wan-WendnerFranz L. DickertRonald MarquezFreddys Beltrán GonzálezRoosevelt DelanoFrédéric DumurRoozbeh Abedini-NassabFrederieke LangerRosa María García SalvadorFrederik KotzRosaria BrunoFredric M. MengerRosario González MotaFriederike SchmidRose-Marie LatonenFuat TopuzRossella ArrigoFumio NaritaRossella SuraceGábor VargaRoxana JijieGabriel Aguirre-ÁlvarezRoxana RacoviceanuGabriel FurtosRoxana RuseckaiteGabriela DudekRuben ForestiGabriela RapeanuRubén González NúñezGabriela RosaRuben J. Sanchez RodriguezGabriela Wiosna-SałygaRubia Figueredo GouveiaGabriele CarulloRucha NatuGael Y. RochefortRuchir PriyadarshiGaetano IsolaRudi SeitzGalder KortaberriaRudinei FiorioGalina V. KurlyandskayaRudolf HolzeGamal A. El-HitiRudolf KieferGanesh Dattatraya SarataleRudolf PfaendnerGanesh SarataleRui ChangGang WeiRui M. S. CruzGarry KerchRui MinGary TepperRui S. CostaGennaro AgrimiRui YuanGennaro GentileRuibin WangGeoffrey MitchellRuimeng ZhangGeorge KenanakisRun-Sheng LinGeorge VerrosRuoxing WangGeorge VoyiatzisRushdan Ahmad IlyasGeorge Z. KyzasRuslan R. KashapovGeorgi G. GochevRusso EleonoraGeorgia LainiotiRyo IshiharaGeorgios BokiasRyohei IshigeGeorgios EleftheriadisSabata MartinoGeorgios K. KoulinasSabina AbbrentGeorgios PappasSabina ZiembowiczGeorgy LazorenkoSabine MatysGerald FranzSabornie ChatterjeeGerhard SinnSabrina CarroccioGert DorenbosSabyasachi GaanGhanashyam AcharyaSachin NavaleGheorghe NechiforSadanand PandeyGiacomo DerchiSagrario Torres-CartasGiada Lo ReSaifullah BulloGianluca CicalaSaikat Sinha RayGianluca TondiSajjad Husain MirGianluca ViscusiSaleem IqbalGianluigi AlbanoSalim HizirogluGi-Byoung HwangSalma BilalGimyeong SeongSalmabanu LuharGiorgio LucianoSalvatore GalloGiorgio SperanzaSalvatore VerreGiovanni LucchettaSameer Ahmed AwadGiovanni N. RovielloSamir KhatirGiovanni VozziSamy MadboulyGirolamo CostanzaSana UllahGisbert RiessSánchez Poblete Julio AntonioGiulia ScaletSándor GóbiGiuliano ZanchettaSándor KékiGiuseppe CavallaroSándor Kunsági-MátéGiuseppe Cesare LamaSandra CruzGiuseppe CinelliSandra PaszkiewiczGiuseppe CirilloSandra PinaGiuseppe FlorioSandro LongoGiuseppe LazzaraSang Woo KimGiuseppe PellicaneSangram SamalGiuseppe PortaleSangwon KimGiuseppe StortiSanja Kostadinovic VelickovskaGiuseppe Trusso SfrazzettoSanjairaj VijayavenkataramanGloria Huerta-AngelesSanjeev KumarGonzalo Martínez BarreraSanju GuptaGonzalo VillaverdeSanoj RejinoldGopinathan JanarthananSantiago FerrandizGoshtasp CheraghianSantosh KumarGraeme MoadSara I. AbdelsalamGrant WebberSara LiparotiGrażyna AdamusSara PerteghellaGražyna Simha MartynkováSarang GumfekarGregory KowalskiSaranshu SinglaGreta AdamczykSascha RohnGrzegorz BudzikSathish Sundar Dhilip KumarGrzegorz DombekSathiyaraj KandhasamyGrzegorz KowalukSathiyaseelan AnbazhaganGrzegorz ŁapienisSatish ToteyGrzegorz LesiukSatoshi YokoseGrzegorz Ludwik GolewskiSatyawan NaganeGrzegorz SchroederSaulius GrigaleviciusGuadalupe Virginia Nevárez-MoorillónSaulius JuodkazisGuangchao ZhengSavvas VassiliadisGue-Hyun KimSawsan A. ZaitoneGuido La RosaSayan GangulyGuido PanzarasaSayyeda Marziya HasanGuilherme FerreiraSebastian DahleGuillermo AhumadaSebastian SchwamingerGuillermo J. CopelloSebastian SławskiGundula Gesine Schulze-TanzilSebastiano GarroniGundula HelschSeigou KawaguchiGunjae JeongSeisuke AtaGuo Dong GohSekar VijayakumarGuo Liang GohSelvin P. ThomasGuoli TuSenem KamilogluGuoying DongSenthil KaliappanGurjot S. DhaliwalSeongwoo RyuGuru Raghavendra ValicherlaSergei V. KostjukGurunathan ThangavelSergey KonovalovGuruprakash SubbiahdossSergey LarinGustavo Cabrera-BarjasSergio CiceroGustavo FernandesSérgio Frascino Muller De AlmeidaGyorgy DeakSergio MadurgaGyorgy SzekelySergio SambataroHaci BaykaraSergiu CoseriHadi RastinSerguei Alejandro-MartínHae-Hyoung LeeSeth DarlingHaibao LuSeyed Ali GhahariHai-Chou ChangSeyed Amid TahamiHaile Fentahun DaegeSeyedhosein PayandehHaipeng YuShabi Abbas ZaidiHaishun DuShahi RejahHaitao CuiShahin HomaeigoharHaitao ZhaoShang-Ta WangHamada ElwanShaobo ZhouHamdy F. M. MohamedShaochuan FengHamed Aghajani DerazkolaShaofeng LiuHamid Reza TaghiyariShaohua JiangHammad KhalidShaojian HeHamouda M. MousaShaolin ZhangHanako AsaiShaolong SunHanfei MeiShaoping QianHani SalimShaoyang LiuHanieh KargarzadehShaoyang WangHany AbdoSharifu UraHany M. R. Abdel-LatifShashi Kant BhatiaHany MareiShazid Md. SharkerHaroutioun AskanianSheng-Heng ChungHarsh VardhanShereen M. S. Abdel-HamidHasan SadeghifarSherif MehannyHassan A. AlshahraniShiaowei KuoHatice MutluShiao-Wei KuoHe LiShingo YokotaHéctor Aguilar-BoladosShin-ichiro TohmuraHéctor Santiago-HernándezShintaro SukegawaHeidari Behzad ShiroudShipeng WenHelena FelgueirasShisong RenHelinando Pequeno De OliveiraShiuhchuan HerHemraj M. YadavShiuh-Tzung LiuHenri VahabiShogo TakamukuHenrique Almeida SantanaShotaro NishitsujiHenryk KaniaShou-Heng LiuHeriberto Perez-AceboShreya Roy ChoudhuryHideharu MoriShuai LiangHideki Amii Shuang SongHideki HayashiShu-Chuan LiaoHideto TsujiShu-Hsien HuangHilal KhanShujun ChenHimanshu KathuriaShu-Kai YehHimanshu Sekhar JenaShuo Park LinHiromu KondoShuvra SinghaHirosato MonobeShuye ZhangHiroshi YoshiharaShyong Siow KimHiroyuki MuramatsuSiddhartha DattaHo Dong KimSilva JoãoHoichang YangSilvana AlfeiHong ZhangSilvia Nair GoyanesHong ZhaoSilvia TabacchioniHongxi LuoSimona BadilescuHongyu ZhengSimona CăprărescuHoon Suk RhoSimona Gabriela BungauHoria-Eugen PorteanuSimona MartinottiHossam El-Din SallamSintu RongpipiHossein Cheraghi BidsorkhiSipei LiHossein EslamiSithiprumnea DulHossein HaghighiSixun ZhengHouwen PanSławomir KulaHsiharng YangSneh PuniaHsiu-Wen ChienSneha SamalHsuanling KaoSnezana Ilic-StojanovicHu LiSnezana NenadovicHu ZhangSobhi GomhaHuanda ZhengSofia M. MorozovaHui SunSofia RangouHuiqun WangSohail YasinHui-Yu ChenSoheil GohariHung-Yin LinSomen K. BhudoliaHusam YounesSoňa RusnákováHwan D. KimSong ZhangHyeonyeol JeonSónia CarabineiroHyun Jin ParkSónia SimõesHyun KimSonja Herres-PawlisIacopo BenesperiSotirios G. StavropoulosIan WymanSpiros VlahopoulosIgnacio JessopSpyros PapageorgiouIgnacio RintoulSridhar VadahanambiIgnazio BlancoSriram Gopu GopuIgor E. GolubStamatis KarlosIgor E. SoshnikovStanislaw KlosowiczIgor S. SirotinStanislaw SlomkowskiIgor SedovStavros TsantzalisIgor SlivacStefan KöstlerIgor V. KhudyakovȘtefan ȚăluIhsanullah IhsanullahStefan WolnyIlenia FarinaStefan ZechelIlker S. BayerStefania CantoreIlkeun LeeStefania PragliolaIlya A. MorozovStefano CrespiIlya G. ShenderovichStefano FioriIlya UlasovStefano LeporattiIman YazdiStefano PaganoImena RexhepiStefan-Ovidiu DimaImran FarooqStepan PodzimekImre KissStéphanie AndradeIn Woo CheongStephanie WillerthIndranil RoyStephen AmiandamhenInese FilipovaStephen WalleyIngars ReinholdsSteven W. BucknerIoana DemetrescuStylianos ExarhopoulosIoana StanculescuSuathong GohIoanna NtaikouSubramanian SundarrajanIoannis BratsosSudip MondalIoannis KoutselasSudipta PanjaIoannis ManolakisSuguna PerumalIoannis SamarasSuhana KotingIolanda De MarcoSumant DwivediIolanda FrancoliniSu-Mi HurIonela Andreea NeacsuSumit Sinha RayIonela NicaSumita GoswamiIonut-Cristian RaduSuneel KumarIrena Kratochvilova Sung Woo SohnIrene Buj-CorralSung Woon ChaIrene S. FahimSung-ae SonIrina SulaevaSunghan KimIrina YushinaSunhee LeeIsamu AkibaSunil K. SharmaIsha MutrejaSunil Kumar BodaIsrael GonzálezSunil Kumar Lindström RamamoorthyIstvan LaszloSunil Kumar RamamoorthyIstvan SzaboSunil Kumar SharmaIuco Castro Da Silva IgorSun-Mou LaiIurii VozniakSunpreet SinghIva RezićSupachok TanpichaiIva SovadinováSupandeep Singh HallanIvan A. ParinovSurendra Krushna ShindeIvan FortelnýSurendra ThapaIvan GitsovSurya Veerendra Prabhakar VattikutiIvan HevusSuryadi IsmadjiIvan RužiakSusanne K. WocheIvan SkvortsovSuzane Cristina PigossiIvana PantelicSuzanne MartinIvana Salopek ČubrićSvemir RudićIvo ArosoSvetislav SavovicIwona KarbownikSwarna JaiswalIwona RykowskaSwarup RoyIwona ŚcibiszSwee Leong SingIyyakkannu SivanesanSylwia GolbaIzabela Barszczewska-RybarekSzetsen LeeIzabela IrskaSzilvia KlebertIzabela ŚliwaTae Joon KwakJacek FalTaekeun OhJacek NowaczykTahir NooraniJacek WychowaniecTairong KuangJack SkinnerTakahiko NakaokiJacob SheryTakahiro GunjiJacobo Hernández-MontelongoTakahiro KannoJacopo TirilloTakahiro SatoJadwiga LaskaTakahito ItohJae Bem YouTakaki KanbaraJae Yong KimTakashi AzumaJaehan JungTakashi KajitaniJaehan LeeTakayuki TsukegiJae-Hong ChoiTakeshi AoyagiJaejong LeeTakuo SakonJae-Myung RyuTakuya KatashimaJagoba IturriTamara D. Lazarević-PaštiJahir Orozco HolguínTamás HaideggerJaime EscalanteTamerlan T. MagkoevJaime Javier JuárezTan Jin SooJaime ViñaTanaka YoJakub Dalibor RybkaTaner SarJakub HaberkoTanmoy MukhopadhyayJakub MatusiakTanveer Ahmad TabishJamal KhatibTao HuangJames ChowTao XuJames Kit-hon TsoiTao ZhangJames MollerTao-Hsing ChenJamieson BrechtlTarek M. Abou ElmaatyJan BrodaTatjana ŠkrbićJan KrmelaTatsiana MikulchykJan MacutkevičTe I. LiuJan PaczesnyTeng ChiJan SzadkowskiTeng-Chun YangJana SedlaříkováTeodora StaicuJang KoTeofil JesionowskiJanusz CieślińskiTeresa BasinskaJanusz KluczynskiTeresa RegueiraJanusz KozakiewiczTetsuo YamaguchiJanusz W. SikoraThai Thanh Hoang ThiJaroslav KousalThanh-Canh HuynhJasleen Kaur BindraThanh-Danh NguyenJasmine SinhaThashree MarimuthuJason Thomas DuskeyThemistoklis KabanosJasvir DalalTheodoros ManourasJaved AhmadTheodoros RousakisJavid HussainThi Tuong Vy PhanJavier Fernández-RodríguezThiagarajan MadheswaranJavier IllescasThomas DippongJayachandra Hari MangalaraThomas GkourmpisJayachandran VenkatesanThomas HellwegJayalakshmi AyyavooThomas J. SmithJea Uk LeeThomas KeanJean François DrilletThomas LuxbacherJean-François GohyThomas SchnabelJechan LeeThomas SchweizerJeevithan ElangoThore RohwerderJelena GulicovskiTianlong LiJem-Kun ChenTianpeng DingJennifer PattersonTiciane Santos ValeraJens SmiatekTien Duc PhamJeong Jae WieTim OsswaldJerzy BochniaTimothy HanksJerzy J. ChruścielTimur Sh. AtabaevJerzy J. LangerTitus A. JennyJerzy SmardzewskiTiziana FuocoJerzy Wojciech MietelskiTo NgaiJessika BertacchiniTomasz JaroszJesús Fernando Ayala ZavalaTomasz KowalczykJesús-María García-MartínezTomasz KoziorJhantu Kumar SahaTomasz KrystofiakJia WeiTomasz NiedzielaJiahao ChenTomasz PanczykJiajia XueTomasz RybickiJialong ShenTomasz RydzkowskiJian WuTomasz SzymborskiJian YuTomasz TrzepiecinskiJiancheng LuoTomasz ŻelazińskiJianfeng LiTomaž PepelnjakJianlei WangTong DengJian-Wen WangTonino TrainiJianxun DingToru KanbayashiJian-Zhang ChenToshihiro KanekoJiayi YuToshihiro ShimadaJiayuan JiToshihiro TakashimaJie LuoToshiyuki KawaiJifeng GaoTraian ZaharescuJigneshkumar PatelTrevor D. RapsonJin GaoTruong Phuoc NghiaJin LiTsung-Rong KuoJin MotoyanagiTuan Anh NguyenJing ChenTurgay SeçkinJing GuoTzu-En LinJingfa LiUgo CarusoJingjing GaoUgo LafontJinguang CaiUlises Páramo-GarcíaJingyao SunUlphard Thoden van VelzenJing-Yun WuUlrich GieseJin-Jia HuUmair BaigJinming HuUmapathi ReddicherlaJinming LiuUmberto PriscoJinxin LiUrszula BazylinskaJinyang XuUrszula Krupa-KozakJiři HorskyUrszula StachewiczJiří Jaromír KlemešUwe LappanJiří SkládankaVagelis HarmandarisJitang FanValdecir Antonio PaganinJitka MohelnikovaValentina DomeniciJi-Won ParkValentina MazzantiJoachim HeinValentina SessiniJoachim StorsbergValentina VasilevskayaJoachim StumpeValeria PasciuJoana C. AntunesValerio GrazianiJoanna BrzeskaValery E. TarabankoJoanna CzechowskaVamsee VadlamudiJoanna CzwartosVan-Chuc NguyenJoanna GościańskaVan-Duong DaoJoanna KrakowiakVanessa AntunesJoanna KujawaVarsha D. PhadtareJoanna LiszkowskaVarun VohraJoanna MystkowskaVasil D. SimeonovJoanna OrtylVasile CojocaruJoanna Paciorek-SadowskaVasileios C. KapsalisJoanna RaczkowskaVasilica PopescuJoanna RydzVassiliki KontargyriJoanna RyszkowskaVassilios SikavitsasJoannis K. KallitsisVedyagin AlekseyJoão Miguel Marques Dos SantosVeerakumar ArumugamJoão Nunes-PereiraVera V. BudaevaJoão Paulo LealVerónica Montes GarcíaJoão Paulo Saraiva MoraisVeronica San MiguelJoao Paulo TribstVeronica VespiniJoão SilvaVicente Espinosa-SolisJoao TedimVictor AtuchinJoaquim C. G. Esteves Da SilvaVictor IzraylitJoaquín C. García-MartinezVíctor Javier Cruz-DelgadoJohanna MajoinenVíctor Manuel Pérez PuyanaJohannes LützenkirchenVijay Kumar ThakurJohn HalloranVi-Khanh TruongJohn WilliamsViktor Korzhikov-VlakhJoji OkazakiViktor SavovJolanta EjflerViktoria MannheimJolanta KowalonekViktoria MilkovaJolanta SzymańskaVincent ChanJon MaizVincenzo BellantoneJonas AlexandreVincenzo TufarelliJong Won KimVincenzo VillaniJong-Ning AohVineet KumarJong-Seok ParkVinod V. T. PadilJordi Farjas SilvaVioleta MelinteJordi FraxedasViorica MusatJordi PoaterVirginija JankauskaiteJörg FitterVitalii I. SysoevJorge Alonso Uribe-CalderónVitaly A. MorozovJorge CernaVito BasileJorge Cesar MasiniVito GiganteJorge EscorihuelaVivek SaraswatJorge PeixinhoVivek TrivediJorge SilvaVjaceslavs LapkovskisJose A. Jofre-RecheVladimir Eugenievich YudinJose Adrian Martinez-GonzalezVladimir I. LozinskyJosé Antonio Luceño SánchezVladimir M. Gun’koJosé Bonilla-CruzVladimir PopovJosé Claudio CaraschiVladimir VelikanovJosé Cleiton Sousa Dos SantosVlassios LikodimosJose CornejoVolker AltstaedtJosé Enrique Torres VaamondeVolker SchöppnerJose Gamez-PerezVu Khac Hoang BuiJose Garcia De La TorreWahid FerdousJosé Ginés Hernández-CifreWai Yee YeongJosé J. Arroyo GómezWalter EeversJose Luis De PazWalter Remo CaseriJosé Luis Rivera-ArmentaWanda SikorskaJosé María Palacios-SantanderWanhe HuJose Ramon LeizaWan-Ting ChenJose Roberto BauerWanting NiuJosef JůzaWarintorn ThitsartarnJosefina PonsWarren BluntJosé-Luis MaldonadoWaruna DissanayakaJoseph C. Furgal Wasuke MoriJoseph MarcinkoWei CuiJosip BrnićWei Long NgJosu OrtegaWei ShaoJosy A. OsajimaWei WuJózef HaponiukWei YangJr-Jeng RuanWei ZhaoJu DongWeibing ZhongJuan C. CruzWei-Dan DingJuan Carlos Cabanelas ValcárcelWei-Dong HeJuan Carlos Campos RubioWeihong WangJuan Hernández-ÁvilaWeimin HuangJuan J. FreireWen ZhangJuan José López-CelaWenbin ZhouJuan Monzó-CabreraWen-Cheng ChenJuan Rodrigo Vélez-CorderoWenjing LinJuan Tolosa BarrileroWen-Ren JongJue-Hyun LeeWensheng WangJuhyun ParkWen-Ta SuJui-Teng LinWen-Ten KuoJui-Yang LaiWenxin FuJulia Alvarez-MalmagroWerner PauerJulia Pribyl HamWesley Lyeverton Correia RibeiroJulia RevueltaWidiastuti SetyaningsihJulien FérecWilman CarrilloJulio A. Seijas VázquezWirginia TomczakJun KoyanagiWitold ZukowskiJun LuoWoei Jye LauJun NieWojciech FabianowskiJun XuWojciech KujawskiJune-Seok HeoWojciech WalachJun-Ichi KadokawaWojciech WielebaJunjie LiWubin YingJunjie WangWujie ZhangJunsheng LiXabier GómezJunyi LiuXavier ColomJuran KimXia LiJure ŽigonXiaming FengJurgita MalaiškienėXian Hu LiuJustyna KozłowskaXiang LiJustyna Mozejko-CiesielskaXiang WangJustyna SulejXiangguo TengJu-Won JeonXiangling JiJwala RenukuntlaXiangzhong ChenK. KaruppasamyXianjie LiuK. KenryXiaofeng WangKadir BilisikXiaohang SunKadir OzaltinXiaohong WangKaiyang YinXiao-Kun OuyangKaiyue ZhangXiaolong JiaKalavathy RajanXiaomin ZhuKállai NikolettXiaoning ZhangKamal Hany HusseinXiaoqun ZhuKamil KaminskiXiaorui ChenKamila Rybczyńska-TkaczykXiaoxia LeKamol DeyXiaoyong WangKamran KhanXiaoyu HuangKar Wey YongXiaozhong QuKarol SzubertXin ChengKarol WolskiXin WuKarolina CysewskaXinda LiKarolina KraśniewskaXinyan DaiKarolina LabusXinyu ZhangKarolina PyciaXiong GongKarthik TappaXu ZhangKartik BeheraXubiao LuoKaryn JarvisXuezhen WangKashif IrshadYaewon ParkKashma SharmaYahya ChoonaraKasper EerselsYahya RharbiKasturi Joshi-NavareYan HanKasza GyörgyYan LiKatarina Dimic-MisicYan WangKatarina MonkovaYang HuKatarína ValachováYang LiuKatarzyna JedynakYang LuKatarzyna KnozowskaYang TongKatarzyna LewandowskaYang-Chun LeeKatarzyna PiotrowskaYang-Fei ZhangKatarzyna PobiegaYan-Hsiung WangKatarzyna Pogorzelec–GlaserYanhua LuoKatarzyna RutkowskaYanwei LiKatarzyna SkórczewskaYanxiao MaKatarzyna StaszakYao WangKatarzyna TandeckaYaodong LiuKateryna FatyeyevaYasir HaleemKatsuhiko ArigaYasumoto NakazawaKavita SharmaYe ShaKazem Reza KashyzadehYeau-Ren JengKazuhiro P. IzawaYee Sin AngKazuya SaitoYexiang TongKe QuYi ChenKeith RochfortYi-Chun ChenKejia YangYifan DongKelly Johana Figueroa LópezYifan LiKelly VeloniaYifei JinKen TeraoYifeng CaoKening LangYijun ZhangKenji ItakaYiming WangKeon-Soo JangYing TieKetan M. RanchYing YangKeum Hwan ParkYingfeng YuKevin FreedmanYing-Guo ZhouKhaled ElsaidYingrui ShangKhaled GiasinYingtao LiuKhaled KhalilYingxiang LiuKhalid ElwakeelYin-Hwa ShihKhalid LamnawarYi-Ting ChiangKhalisanni KhalidYi-Tsu ChanKiat Hwa ChanYiwei TianKinga KorniejenkoYiwen LiKira E. VostrikovaYogendra MishraKirstin VachYohei KotsuchibashiKit Leong CheongYong Chae JungKiyoshi FujisawaYong RenKlaus BöningYong YangKlaus HolschemacherYongfeng GaoKlaus StöweYonglai LuKlemen BohincYongsheng LengKoichi ShinkaiYoshimi SonodaKoichiro ShiomoriYoshinori ArisakaKoizumi ToshioYoshito IkadaKojiro UetaniYoung Hoon SongKonstantinos A. LioliosYoung-Wan KwonKonstantinos DimosYounkee PaikKonstantinos NtetsikasYousuf MohammedKonstantinos TsongasYu Kyoung RyuKorzhikova-Vlakh EvgeniaYu SekiguchiKozo MatsumotoYu ZhangKrishanu RoyYuanhao GuoKristine V. AleksanyanYuankun LinKrystian Leonard ChrzanYuanzuo LiKrzysztof ChwastekYuching LeeKrzysztof FormelaYu-Chun KungKrzysztof GrzelakYuecheng ZhouKrzysztof KukielkaYuhoon HwangKrzysztof MoraczewskiYu-Hsuan ChiaoKrzysztof PielichowskiYuichi IshidaKrzysztof SkrzypkowskiYuichiro NoiriKrzysztof WilczyńskiYukari OdaKsenia KirichenkoYukihiro MatsumotoKumari KavitaYuliar FirdausKun LiangYuliya SilinaKun ZhouYun-Che WangKunal MishraYungjin WengKuniaki NagamineYuri VoznyakKunlun HongYurij StetsyshynKurki Nagaraja BharathYury A. SkorikKyung Hye JungYusuke HiejimaLacramioara PopaYusuke YamadaLadislav ReinprechtYuushou NakayamaLage Miguel Angel PrietoYu-Wei ChengLai Li SzeYuzhan LiLaibao ZhangYves GourinatLajos NagyZahra Mazrouei SebdaniLalith B. Suragani VenuZaida OrtegaLara PerrinZakariya Y. AlgamalLászló AlmásyZbigniew BrytanLászló JanovákZbigniew NadolnyLaura AliottaZbyšek PoselLaura BoggioniŽelimir JelčićLaura BulgariuZeljka PersuricLaura ChronopoulouZelong XieLaura Cristina RusuZengqian ShiLaurence LecampZhaohui HuangLavinia Cosmina ArdeleanZhen ZhangLazaros TzounisZhenyi ZhangLeela RakeshZhenyu LiLei ChenZhi LiuLei LiZhibo YangLei TaoZhihuan WengLei WangZhiling HouLei ZhaiZhiqiang ShenLei ZhuZhiyuan QianLeire Ruiz-RubioZhongkai ChengLejeunes StéphaneZhongzheng ZhangLenuta ProfireZhujin YangLeonard AtanaseZhuoran MaLeonel Jorge Ribeiro NunesZian JiaLeonel Paredes-MadridZihao OuLeonzio FortunatoZilu WangLesław JuszczakZiwen JiangLeszek ŁatkaZlatan DenchevLeto-Aikaterini TzivelekaZohaib KhurshidLevente CsokaZoi TerzopoulouLi FuZoltán Ádám TamusLi LiZongliang WangLi LuZoran DomitranLiang HongZoran DžolićLiangting SunZoran ZekovićLibing ZhengZuzana BenkováLidiia KolzunovaZuzanna Żołek-TryznowskaLidija Gradišnik



